# The Use of Interferon Gamma Inducible Protein 10 as a Potential Biomarker in the Diagnosis of Latent Tuberculosis Infection in Uganda

**DOI:** 10.1371/journal.pone.0146098

**Published:** 2016-01-15

**Authors:** Irene Andia Biraro, Simon Kimuda, Moses Egesa, Stephen Cose, Emily L. Webb, Moses Joloba, Steven G. Smith, Alison M. Elliott, Hazel M. Dockrell, Achilles Katamba

**Affiliations:** 1 Makerere University College of Health Sciences, Kampala, Uganda; 2 Medical Research Council/Uganda Virus Research Institute, Uganda Research Unit on AIDS, Entebbe, Uganda; 3 Department of Clinical Research, London School of Hygiene & Tropical Medicine, London, United Kingdom; 4 Department of Infectious Disease Epidemiology, London School of Hygiene & Tropical Medicine, London, United Kingdom; 5 Department of Immunology and Infection, London School of Hygiene & Tropical Medicine, London, United Kingdom; The Catholic University of the Sacred Heart, Rome, ITALY

## Abstract

**Background:**

In the absence of a gold standard for the diagnosis of latent tuberculosis (TB) infection (LTBI), the current tests available for the diagnosis of LTBI are limited by their inability to differentiate between LTBI and active TB disease. We investigated IP-10 as a potential biomarker for LTBI among household contacts exposed to sputum positive active TB cases.

**Methods:**

Active TB cases and contacts were recruited into a cohort with six months’ follow-up. Contacts were tested for LTBI using QuantiFERON^®^-TB Gold In-Tube (QFN) assay and the tuberculin skin test (TST). Baseline supernatants from the QFN assay of 237 contacts and 102 active TB cases were analysed for *Mycobacterium tuberculosis* (MTB) specific and mitogen specific IP-10 responses.

**Results:**

Contacts with LTBI (QFN^+^TST^+^) had the highest MTB specific IP-10 responses at baseline, compared to uninfected contacts (QFN^-^TST^-^) p<0.0001; and active cases, p = 0.01. Using a cut-off of 8,239 pg/ml, MTB specific IP-10 was able to diagnose LTBI with a sensitivity of 87.1% (95% CI, 76.2–94.3) and specificity of 90.9% (95% CI, 81.3–96.6). MTB specific to mitogen specific IP-10 ratio was higher in HIV negative active TB cases, compared to HIV negative latently infected contacts, p = 0.0004. Concentrations of MTB specific IP-10 were higher in contacts with TST conversion (negative at baseline, positive at 6-months) than in those that were persistently TST negative, p = 0.001.

**Conclusion:**

IP-10 performed well in differentiating contacts with either latent or active TB from those who were uninfected but was not able to differentiate LTBI from active disease except when MTB specific to mitogen specific ratios were used in HIV negative adults. In addition, IP-10 had the potential to diagnose ‘recent TB infection’ in persons classified as having LTBI using the TST. Such individuals with strong IP-10 responses would likely benefit from chemoprophylaxis.

## Introduction

It has been estimated that approximately one third of the world’s population is latently infected with *Mycobacterium tuberculosis* (MTB) [1]. Optimal diagnosis of latent tuberculosis infection (LTBI) should be aimed at persons with the highest risk of progression to active tuberculosis (TB) disease [2, 3] who will benefit from prompt treatment, and contribute to reduction in transmission of the infection to others [4]. There is no gold standard test for the diagnosis of LTBI [5, 6] and many are still using the tuberculin skin test (TST), despite its limitation of false positive results secondary to prior exposure to environmental mycobacteria or Bacillus Calmette–Guérin (BCG) immunisation [7]. The TST is also influenced by inter or intra reader variability and immunosuppression status of the host [8]. The other tests used to diagnose LTBI are the interferon gamma release assays (IGRA). These have higher specificity than the TST [9] because they use antigens from the RD1 region of the mycobacterial genome [10] such as early secreted antigenic target 6 (ESAT-6) [11, 12] and culture filtrate protein 10 (CFP-10) [13], proteins which are lacking in BCG [14, 15] and in many pathogenic environmental mycobacteria including *Mycobacterium avium* [16].

There is good correlation between TST and IGRA in low TB endemic settings [17, 18] but high levels of discordance have been reported in some high TB endemic areas including Uganda [19]. Most of the discordance occurs with individuals having TST negative but IGRA positive results [5], although some studies have reported the reverse [20–22]. The latter discordance has been linked predominantly to BCG [9, 20, 23, 24] which has effects on TST but not on IGRA [18, 20, 25–27]. Other studies report no effect of BCG on TST responses [28, 29]. Both tests are also influenced by the immunosuppression status of the host in particular by immature immune systems, HIV infection, malnutrition, or diabetes mellitus [4].

The TST and IGRA rely on a good delayed-type hypersensitivity (DTH) or T cell immune response following re-exposure to MTB specific antigens, respectively, and are unable to differentiate between latent infection and active disease [5]. This has led to the search for new biomarkers to improve the diagnosis of LTBI. One potential candidate is the interferon inducible protein (IP) 10 (IP-10) [30, 31]. This is the chemokine (CXC motif) ligand 10 expressed by macrophages when stimulated by interferons and other pro-inflammatory cytokines [32–34]. IP-10 induces movement of monocytes and activated Th1 cells to sites of inflammation through its interaction with the CXC chemokine receptor 3 [35, 36]. IP-10 promotes Th1 immune responses by up regulating the expression of IFN-γ and is involved in the DTH immune responses [37, 38]. It has been suggested that production of this chemokine in response to MTB antigens could be used as a marker of infection [39, 40]. Although the use of IP-10 for the diagnosis of LTBI still depends on stimulation with MTB antigens, it is often produced in high quantities, hence providing an amplified signal in immunosuppressed individuals and children [41]. However, some studies have showed low sensitivities of IP-10 in detecting MTB infection among children with active TB disease in a low TB endemic setting [42] and a high TB burden area [43, 44]. IP-10 has also been assessed as a potential biomarker to monitor treatment response to TB medication, whereby IP-10 levels drop between the start of treatment and end of treatment [39].

Our study population had highly discordant TST and IGRA (QuantiFERON^®^-TB Gold In-Tube (QFN)) results which prompted us to explore the use of IP-10 as a potential biomarker for the diagnosis of LTBI. We also assessed the performance of IP-10 in differentiating between latently infected individuals and active TB cases or uninfected individuals.

## Materials and Methods

### Study design and participants

New sputum smear positive TB patients (active TB cases) aged above 18 years and attending the TB clinics at two health centres in Kampala municipality, Uganda, and their healthy household contacts, were enrolled consecutively into a longitudinal cohort. The details of the study settings have previously been described [45]. Both index cases and contacts provided a sample of 3ml of blood for the QuantiFERON^®^-TB Gold In-Tube (QFN), test (Cellestis GmbH (Europe), Hannover, Germany) at baseline and at the end of six months. A TST was placed on the inner surface of the left forearms of the contacts based on the Mantoux method using 2 Tuberculin Units (0.1ml) of RT-23 PPD (Statens Serum Institute, Copenhagen, Denmark) and read after 48–72 hours. The TST was repeated after six months. A positive response was defined as a diameter of the induration area of >5 mm for HIV positive contacts and >10 mm for those who were HIV negative.

### Ethics statement

All index cases and household contacts that were above 18 years of age gave written informed consent to participate in the study. For the household contacts that were below 18 years, the parents and next of kin gave written informed consent on their behalf to participate in the study. Children between 10 and 17 years gave additional written informed assent to participate in the study. The study was approved by both the Makerere University Ethical Review Board and Uganda National Council of Science and Technology.

### IP-10 ELISA assay

Whole blood QFN culture supernatants from contacts and active TB cases at baseline and after six months were analysed for IP-10 responses using Human IP-10 BD OptEIA^™^ ELISA kits (BD Biosciences, USA) [46]. Samples were obtained from all the three QFN tubes (nil (no antigen), antigen and mitogen) and analysed in duplicate wells using 96 well Immulon^®^ 4 HBX microtiter plates (Thermo Scientific^™^, USA). Fifty microliters (1:250) of diluted capture antibody (anti-human IP-10 monoclonal antibody) was added to each well and incubated overnight at 4°C. After washing 3 times, 200 μl of assay diluent (10% fetal bovine serum (FBS) in phosphate buffered saline (PBS)) was added to block the plates and then incubated for 1 hour at room temperature. During this period, standard concentrations of IP-10 were prepared and test samples were diluted in assay diluent (1:8). The plates were then washed 3 times and 50 μl of standard or diluted sample was added to the wells and incubated for 2 hours. After 5 washes, 50 μl of working detector (biotinylated anti-human IP-10 monoclonal antibody + streptavidin-horseradish peroxidase conjugate) diluted 1:250 in assay diluent was added and incubated for 1 hour. The plates were washed 7 times with 30 second to 1-minute soaks and 50 μl of substrate solution added and incubated for 30 minutes at room temperature in the dark. After this, 50 μl of stop solution (2N H_2_SO_4_) was added to each well and the plate optical densities (ODs) were read at 450 nm and 570 nm within 30 minutes using an ELISA plate reader (Biotek). A standard curve was generated from the standards using Gen5^™^ Data Analysis Software and used to convert ODs to IP-10 levels in pg/ml. The range of the standards (sensitivity of the assay) was from 5.8954 pg/ml to 3,000 pg/ml.

The IP-10 values for each individual from the duplicate wells were averaged. The spontaneous (no antigen) IP-10 levels were subtracted from the stimulated (MTB specific antigen) and the mitogen specific data to obtain net MTB specific, and mitogen specific IP-10 responses respectively. The net MTB specific and mitogen specific IP-10 responses were both multiplied by the dilution factor of 8 to obtain the final value.

### Statistical methods

The study was designed to assess the use of IP-10 as a potential biomarker for the diagnosis of LTBI. We divided the study subjects into: uninfected contacts (QFN^-^TST^-^), latently infected contacts (QFN^+^TST^+^), contacts with discordant results (QFN^+^TST^-^ or QFN^-^TST^+^), and the active TB cases. The contacts that had discordant QFN and TST results were excluded from further analysis. Data was analysed using STATA package version 12.

Socio-demographic characteristics (sex, age, HIV status of participant, and presence of a BCG scar among the contacts) of the three remaining groups (uninfected contacts, latently infected contacts, active TB cases) were compared. The Mann-Whitney *U* test was used for comparing continuous characteristics, the chi-squared test for comparing categorical characteristics. Graphs were generated to show the net baseline IP-10 distributions among the three groups and levels of IP-10 were compared with pairwise comparisons made using the Mann-Whitney *U* test. Receiver operating characteristic (ROC) analysis was carried out to determine the sensitivity and specificity of using IP-10 to differentiate 1) individuals with LTBI from uninfected individuals, 2) individuals with active TB from uninfected individuals, and 3) individuals with LTBI from individuals with active TB. Kappa statistics were used for assessing agreement between IP-10 (using the optimal cut-point from the ROC analysis), TST and QFN in differentiating individuals with LTBI from uninfected individuals. Spearman’s correlation coefficient comparing levels of IFNγ generated by the QFN test and levels of MTB specific IP-10 was calculated. Random effects logistic regression models were used to assess how proximity of contact and biological risk factors were associated with the QFN, TST and IP-10 tests in the diagnosis of LTBI.

Among active TB cases, we compared levels of IP-10 between HIV infected and uninfected individuals using the Mann-Whitney U test and multivariable logistic regression. We also assessed the association between TST diameter and levels of IFNγ from the QFN test, and MTB specific IP-10. Lastly, we compared the baseline levels of MTB specific IP-10 among contacts with negative TST/QFN tests at baseline that converted to positive TST/QFN tests at the end of six months and also among contacts with positive TST/QFN tests that reverted to negative TST/QFN tests.

## Results

### Characteristics of study subjects

We enrolled 102 active TB cases and 291 contacts in the main cohort. The median (interquartile range; IQR) number of contacts per active TB case was three (two-four). From this cohort, we analysed whole blood culture supernatants from the QuantiFERON^®^-TB Gold In-Tube assay from 237 contacts and 102 active TB cases at baseline. The contacts were grouped according to their infection status. At baseline, there were 76 uninfected (QFN^-^TST^-^) individuals, 62 latently infected (QFN^+^TST^+^) individuals, and 99 contacts with discordant results (QFN^+^TST^-^ (92) or QFN^-^TST^+^ (7)). The contacts presented with different characteristics from their TB index cases. The contacts were mostly female, of younger ages and HIV negative, while the active TB cases were mostly male, older (because of the inclusion criteria) and were more likely to be HIV positive (Table 1). The uninfected contacts were slightly younger and were more likely to have a BCG scar compared to the contacts with LTBI.

**Table 1:** Participants characteristics.

Variable	Factor	Uninfected n = 66* n (%)	Latent TB n = 62 n (%)	Active index case n = 102 n (%)	p-value
Sex	Female	41 (62)	40 (65)	44 (43)	0.009
	Male	25 (38)	22 (35)	58 (57)	
Age (years)	0–5	20 (30)	13 (21)	0	0.0001
	6–10	15 (23)	4 (6)	0	
	11–15	14 (21)	6 (10)	0	
	16–20	4 (6)	4 (6)	16 (16)	
	21–25	3 (5)	13 (21)	22 (21)	
	26–30	10 (15)	22 (36)	64 (63)	
HIV	Negative	63 (95)	57 (92)	62 (61)	0.0001
	Positive	3 (5)	5 (8)	40 (39)	
Presence of BCG	No	11 (17)	21 (34)		0.02
	Yes	55 (83)	41 (66)		
IP-10 pg/ml	median (IQR)	1,530 (426–3,431)	12,485 (10,565–17,445)	11,725 (7,448–15,183)	0.0001

* 10 missing in uninfected group.

**Fig 1:**
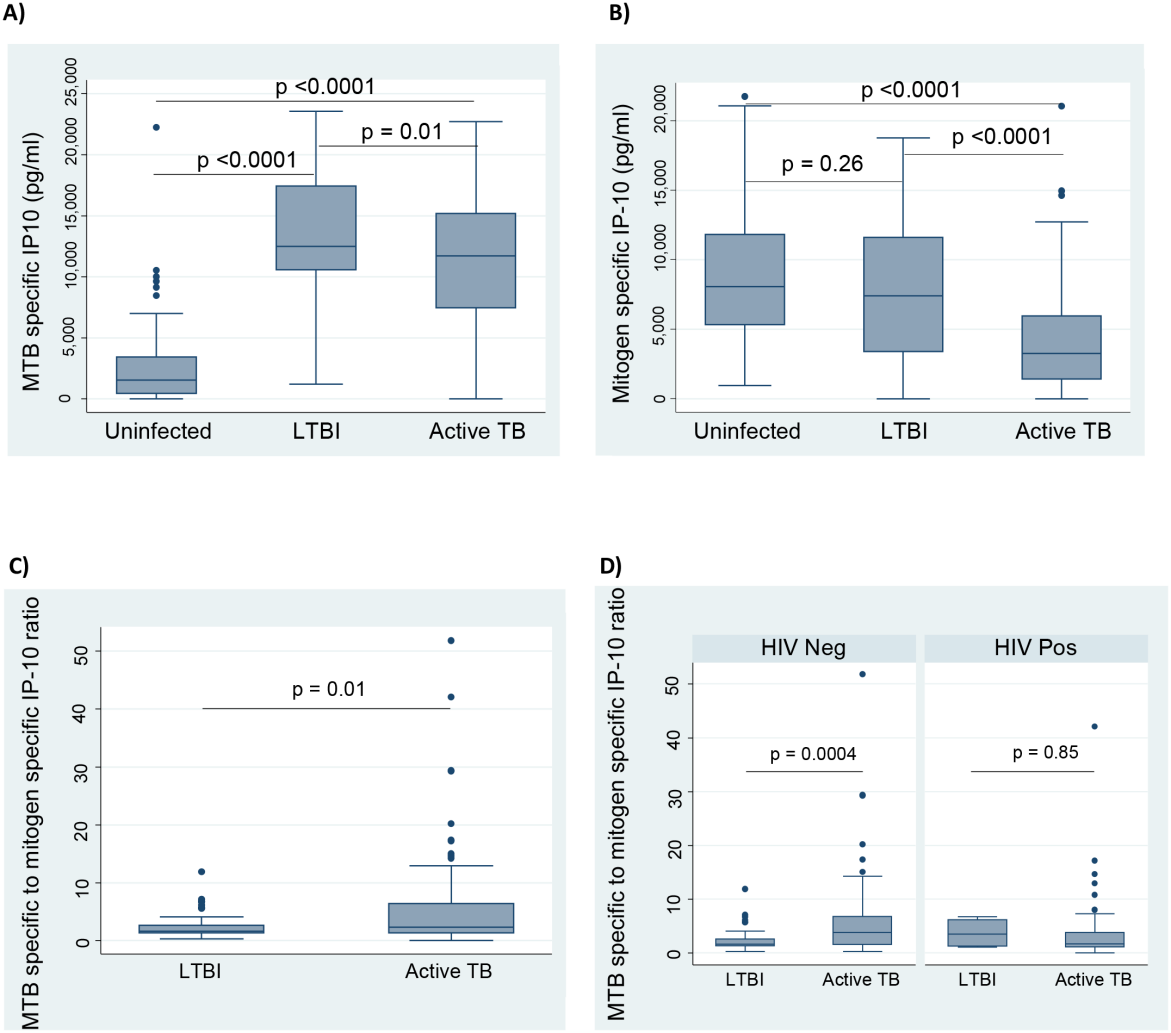
Comparison of MTB specific IP-10 responses, mitogen specific IP-10 responses and MTB specific to mitogen specific ratio among household contacts and active TB cases. Box graphs comparing medians A) MTB antigen specific IP-10 levels, B) mitogen specific IP-10 levels, and C) MTB specific to mitogen specific ratio in uninfected contacts, LTBI contacts and active TB cases. IP-10 was measured in QFN supernatants from uninfected contacts (negative to both TST and by QFN), LTBI contacts (positive in both TST and QFN), and active TB cases at baseline. Baseline uninfected contacts, n = 66; latently infected contacts, n = 62; active TB cases n = 102. Pairwise comparisons were made using the Mann-Whitney *U* test

In response to MTB specific antigens, contacts with LTBI had the highest production of IP-10 (median (interquartile range (IQR)): 12,485pg/ml (10,565–17,445) at baseline, compared to the uninfected contacts 1,530pg/ml (426–3,431) p<0.0001; and to the active cases 11,725pg/ml (7,448–15,183), p = 0.01 (Fig 1A). By contrast, the active TB cases had very low responses to the mitogen (phytohaemagglutinin (PHA)) stimulation (Fig 1B) while contacts with LTBI had similar levels to uninfected contacts. We noted that index cases had high MTB-specific but low mitogen-specific IP-10 responses (whereas contacts with LTBI had high responses to both) we investigated the use of MTB-specific to mitogen PHA specific IP-10 ratios in diagnosing latent and active TB, as well as in discriminating between LTBI and active TB among the three groups. The MTB specific to mitogen specific ratio was slightly higher among the active TB cases (median (IQR)): 5.8 (4.0–7.5) compared to contacts with LTBI 5.3 (-0.2–10.9), p = 0.01 (Fig 1C). A sub analysis was carried out based on HIV status which showed that MTB specific to mitogen specific IP-10 ratio was significantly higher in the HIV negative active TB cases, 3.85 (1.57–6.82) compared to the HIV negative latently infected contacts, 1.62 (1.36–2.62), p = 0.0004 (Fig 1D).

### Potential of IP-10 as a diagnostic marker

**Fig 2:**
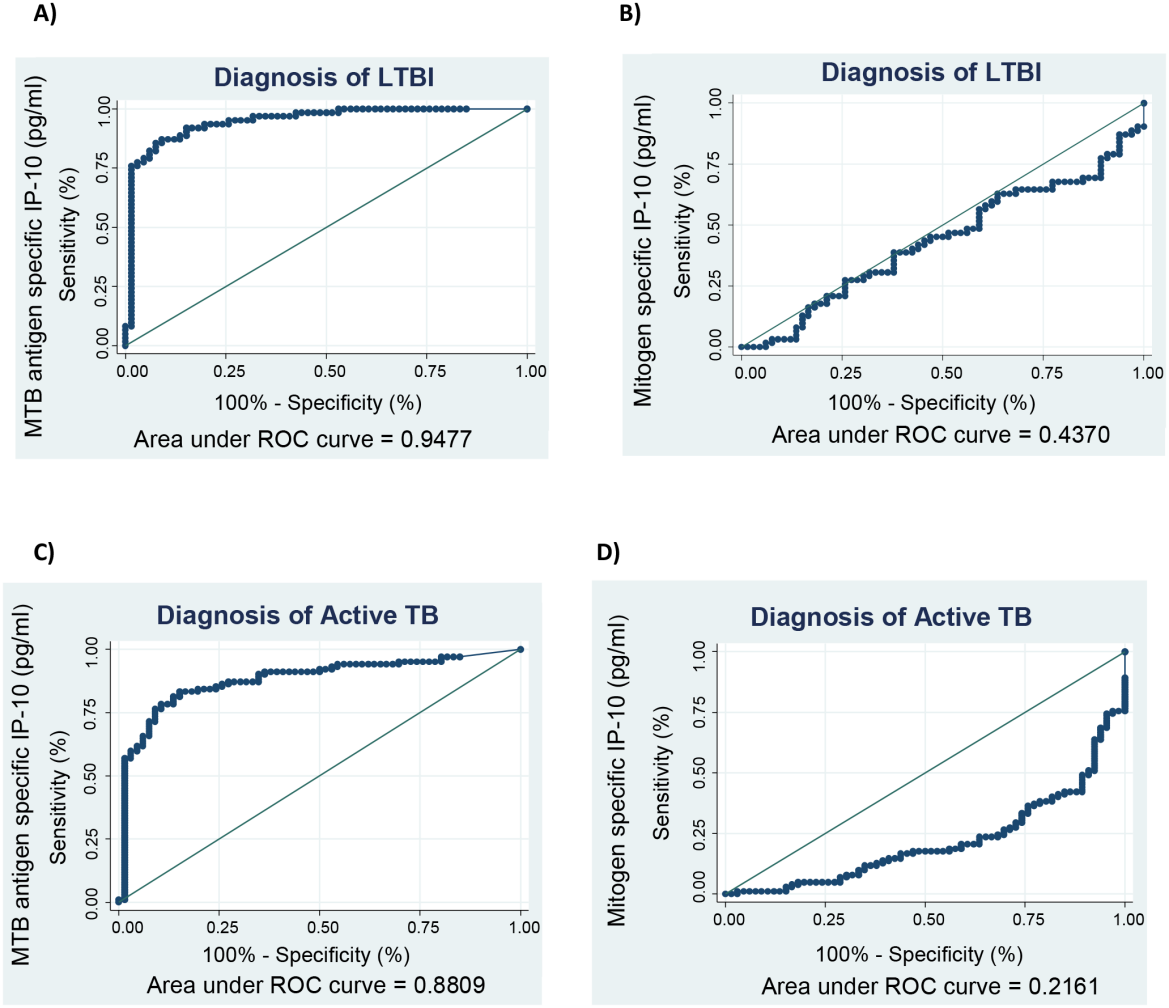
Investigating the diagnostic potential of IP-10 as a biomarker in the diagnosis of latent and active TB. Receiver operating characteristic curves for MTB specific IP-10, mitogen specific, and MTB specific to mitogen stimulated ratio were analysed using the uninfected contacts (negative to both TST and QFN) as the controls and the active TB cases as the diseased group. For the diagnosis of latent tuberculosis (A, and B); for diagnosis of active tuberculosis (C and D).

We assessed the potential of IP-10 as a biomarker for the diagnosis of latent and active TB. At a cut off of 8,239 pg/ml, IP-10 was able to differentiate between the uninfected and latently infected contacts with a sensitivity of 87.1% (95% CI, 76.2–94.3) and specificity of 90.9% (95% CI, 81.3–96.6) (Fig 2A). The mitogen specific responses were not able to diagnose LTBI (Fig 2B). The MTB-specific cut off for diagnosing active TB was lower at 4,858 pg/ml with a sensitivity of 83.3% (95% CI, 74.7–89.9) and specificity of 84.9% (95% CI, 73.9–92.5) (Fig 2C), and the mitogen specific responses still were not able to diagnose active TB disease (Fig 2D). When the MTB specific to mitogen specific ratio was used, it was able to diagnose LTBI at a cut off of 0.88 with a sensitivity of 96.4% (95% CI, 88.8–99.6) and specificity of 89.3% (95% CI, 79.3–95.6). The ratio was also able to diagnose active TB at a cut off of 0.47 with a sensitivity of 96.7% (95% CI, 91.6–99.3) and specificity of 78.7% (95% CI, 66.9–87.8).

### Discriminating LTBI and active TB

**Fig 3:**
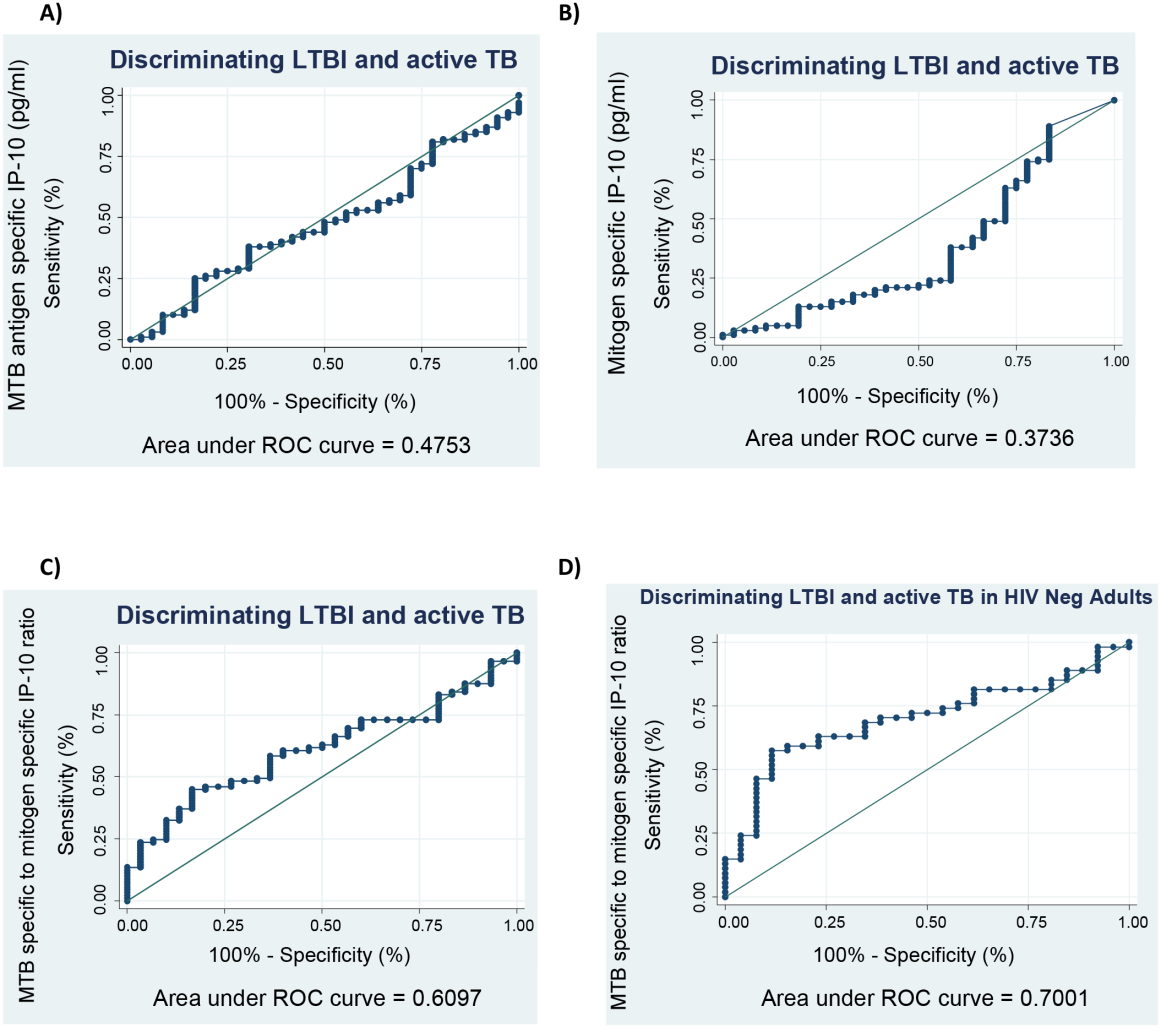
Potential of IP-10 to discriminate LTBI and active TB. Receiver operating characteristic curves to differentiate between latent and active tuberculosis were analysed **f**or the diagnosis of latent tuberculosis using MTB specific IP-10 responses (A), mitogen specific IP-10 responses (B). Adults above 18 years were analysed for MTB specific to mitogen specific IP-10 ratio (C), and a sub analysis among HIV negative individuals (D).

The active TB cases included in this study were adults above 18 years. For this analysis we investigated whether MTB specific IP-10responses, mitogen specific IP-10 responses, and MTB specific to mitogen specific ratios were able to differentiate between latently infected household contacts that were above 18 years and active TB cases. We found that neither MTB specific IP-10 responses nor mitogen specific IP-10 responses were able to differentiate between LTBI infection and active TB disease (Fig 3A and 3B). The MTB specific to mitogen specific ratio was also not able to clearly differentiate between LTBI and active TB with an area under the ROC curve (AUC) (bootstrap confidence intervals (BS-CI) of 0.60 (0.50–0.71) (Fig 3C). However, the MTB specific to mitogen specific ratio was able to differentiate LTBI and active TB in HIV negative adults with an AUC of 0.70 (0.58–0.81) (Fig 3D).

### Prevalence of LTBI

If IP-10 production were used to identify individuals with probable LTBI, based on the cut-off of 8,239 pg/ml, this would give a baseline prevalence of latent tuberculosis among contacts of 55%, compared to 32% with TST, and 65% with QFN. There was a strong agreement between IP-10 and QFN in diagnosis of LTBI (83%, kappa = 0.65), moderate agreement between IP-10 and TST (62%, kappa = 0.27), and poor agreement between QFN and TST (50%, kappa = 0.16).

### Comparing the performance of IP-10 with interferon gamma (IFNγ) in the QFN test

Generally, there was a good correlation between IP-10 levels and IFNγ in the QFN test at baseline among the contacts (Spearman correlation, p-value) of 0.77, p <0.0001, and among the active TB cases (Spearman correlation of 0.63, p <0.0001) (S1 Fig) and this pattern was similar among the three groups: uninfected contacts, latently infected contacts, and the active TB cases (interaction p-value = 0.43).

### Factors affecting response in the diagnostic tests

**Fig 4:**
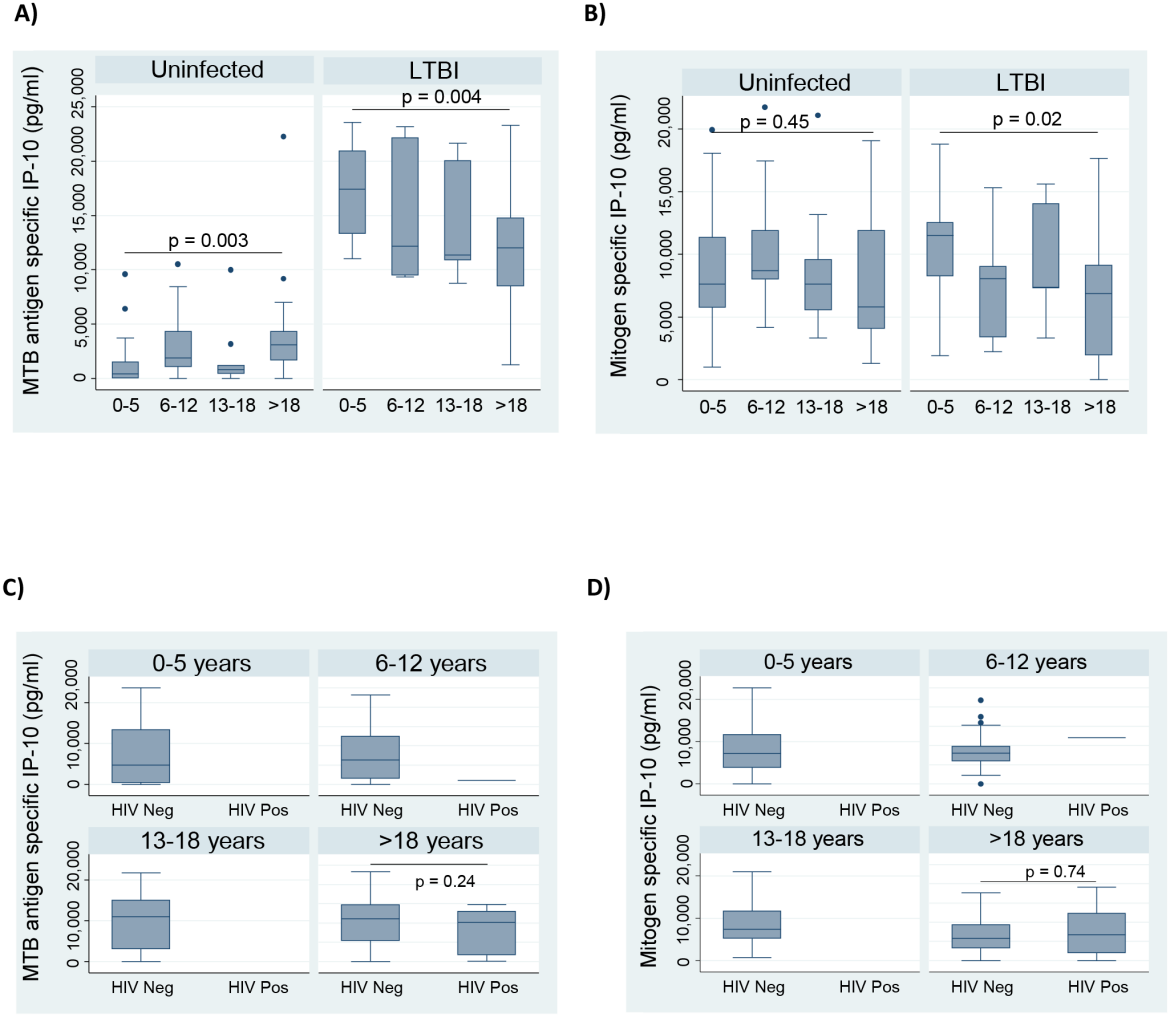
The influence of age on MTB specific IP-10 responses and mitogen specific IP-10 responses among household contacts. Box graphs comparing medians A) MTB antigen specific IP-10 levels, and B) mitogen specific IP-10 levels among the different age groups; C) MTB antigen specific IP-10 levels, and D) mitogen specific IP-10 levels among the different age groups categorized by their HIV infection status. The different age groups included 0–5, n = 55; 6–12, n = 46; 13–17, n = 32; >18, n = 103, and the HIV negative, n = 225; and HIV positive, n = 12. Pairwise comparisons were made using the Mann-Whitney *U* test

The study population included all age groups. The active TB cases were all adults above 18 years of age while the contacts were of all age groups, ranging from 1 year to 70 years. We carried out a sub analysis among the contacts to investigate whether age influenced IP-10 production. The different age groups for this analysis included 0–5 years, 6–12 years, 13–18 years and above 18 years. The youngest contacts (0–5 year) had the lowest MTB-specific IP-10 production among the uninfected contacts and the highest production among the latently infected contacts (p<0.0001) (Fig 4A). Still among contacts with LTBI, the 0–5 age group had the highest mitogen-specific IP-10 responses (Fig 4B).

We assessed putative risk factors among the contacts for association with the different test results (TST, QFN and IP-10) at baseline (Table 2). Gender and HIV status were not associated with a positive result for any test. Increasing age was strongly associated with a positive QFN and IP-10 test but not with TST positivity. QFN and IP-10 were both strongly positively associated with close proximity with the active TB case. The contacts who lacked a BCG scar were most likely to have a positive test result for the TST and IP-10 tests, but there was a weaker association for QFN.

**Table 2:** TB Exposure and Biological Risk Factors Associated with QFN or TST or IP-10 test results among household contacts exposed to active TB cases.

Variable	Factor^¥^	TST n1 = 263		QFN n2 = 277		IP-10 n3 = 237	
		Unadjusted	Adjusted*	Unadjusted	Adjusted*	Unadjusted	Adjusted*
		OR (95% CI); p-value					
Sex	Female (n1 = 154, n2 = 159, n3 = 139)	1	1	1	1	1	1
	Male (n1 = 109, n2 = 118, n3 = 98)	0.75 (0.36–1.56) p = 0.44	1.10 (0.55–2.19) p = 0.77	0.97 (0.52–1.81) p = 0.94	1.53 (0.79–2.96) p = 0.20	0.65 (0.35–1.22) p = 0.18	0.90 (0.46–1.76) p = 0.77
Age (years)	0–5 (n1 = 68, n2 = 68, n3 = 57)	1	1	1	1	1	1
	6–12 (n1 = 49, n2 = 50, n3 = 43)	0.74 (0.20–2.64)	0.79 (0.26–2.40)	0.93 (0.35–2.47)	1.18 (0.46–2.98)	1.66 (0.60–4.55)	2.08 (0.75–5.73)
	13–18 (n1 = 32, n2 = 37, n3 = 35)	0.44 (0.10–1.87)	0.70 (0.21–2.47)	3.21 (1.03–9.97)	4.59 (1.63–12.97)	3.50 (1.15–10.63)	4.65 (1.51–14.32)
	>18 (n1 = 114, n2 = 122, n3 = 102)	3.01 (1.06–8.56) p = 0.01	1.95 (0.79–4.79) p = 0.13	6.22 (2.22–17.42) p = 0.0004	6.15 (2.48–15.25) p = 0.0001	4.22 (1.62–10.99) p = 0.01	3.99 (1.51–10.52) p = 0.02
Relationship to index case	Not first degree (n1 = 85, n2 = 99, n3 = 85)	1	1	1	1	1	1
	First degree (n1 = 178, n2 = 178, n3 = 152)	3.65 (1.58–8.44) p = 0.002	2.29 (0.99–5.30) p = 0.05	2.13 (1.13–4.03) p = 0.01	1.27 (0.64–2.51) p = 0.49	1.37 (0.73–2.58) p = 0.31	0.82 (0.39–1.71) p = 0.60
Proximity to index case	Shared meals (n1 = 50, n2 = 51, n3 = 44)	1	1	1	1	1	1
^**Δ**^ **1 missing n1, 3 missing n2, 2 missing n3**	Cared for index case (n1 = 65, n2 = 69, n3 = 59)	5.20 (1.42–19.0)	3.40 (1.03–11.22)	5.69 (1.93–16.79)	3.40 (1.31–8.84)	5.20 (1.81–14.90)	3.57 (1.19–10.68)
	Slept in same room (n1 = 96, n2 = 104, n3 = 90)	2.96 (0.85–10.32)	2.18 (0.70–6.82)	3.14 (1.22–8.10)	2.38 (1.02–5.54)	4.83 (1.74–13.36)	4.23 (1.39–12.88)
	Slept in same room and bed (n1 = 51, n2 = 50, n3 = 42)	5.08 (1.22–21.03) p = 0.06	3.05 (0.89–10.40) p = 0.19	13.75 (3.63–52.08) p = 0.0007	12.93 (3.78–44.16) p = 0.0004	7.71 (2.29–25.95) p = 0.004	9.42 (2.53–35.10) p = 0.009
Daily duration of contact with index case	<6 hours (n1 = 44, n2 = 44, n3 = 39)	1	1	1	1	1	1
	>6 hours (n1 = 219, n2 = 233, n3 = 198)	3.20 (1.03–9.86) p = 0.04	3.02 (1.02–8.94) p = 0.05	1.18 (0.50–2.78) p = 0.68	1.30 (0.54–3.14) p = 0.54	0.96 (0.40–2.27) p = 0.93	0.89(0.34–2.33) p = 0.82
Presence of a BCG scar	Yes (n1 = 207, n2 = 215, n3 = 188)	1	1	1	1	1	1
^**Δ**^ **2 missing n2, 1 missing n3**	No (n1 = 56, n2 = 60, n3 = 48)	2.92 (1.29–6.60) p = 0.01	2.38 (1.11–5.09) p = 0.02	1.31 (0.57–2.98) p = 0.51	1.24 (0.55–2.77) p = 0.59	2.27 (1.06–4.85) p = 0.03	2.23 (0.94–5.27) p = 0.06
HIV status of household contact	Negative (n1 = 247, n2 = 261, n3 = 225)	1	1	1	1	1	1
	Positive (n1 = 16, n2 = 16, n3 = 12)	3.34 (0.73–15.15) p = 0.11	0.89 (0.24–3.27) p = 0.86	1.22 (0.28–5.16) p = 0.78	0.27 (0.06–1.17) p = 0.08	0.82 (0.21–3.24) p = 0.78	0.36 (0.08–1.59) p = 0.18

^¥^ n1 represents TST, n2 represents QFN, and n3 represents IP-10.

* All the above variables were adjusted for each other in the regression models.

Thirty-nine percent of active TB cases (40/102) were HIV positive, while 5% (12/237) of the contacts were HIV positive. We assessed for the influence of HIV on MTB specific IP-10 responses in the active TB cases and found that there was no significant difference in MTB specific IP-10 levels between the TB cases with HIV infection (mean (95%CI)) 10,631.2 pg/ml (3,934.6–17,327.7) or without HIV infection 13,607.1 pg/ml (12,104–15,110.1) (Mann-Whitney *U* test, p = 0.15). However, the mitogen specific IP-10 responses were lower among the active TB cases that were HIV positive 5,349.2 pg/ml (-1,867.2–12,565.6) compared to those that were HIV negative 7,735.4 pg/ml (6,462.6–9,008.2) (Mann-Whitney *U* test, p = 0.05). A multivariate logistic regression analysis was then carried out among the active TB cases adjusting for age and gender and found no association between HIV and MTB specific IP-10 levels (Adjusted odds ratio (95% CI), p-value) 0.93 (0.10–8.60), p = 0.95. This was repeated for MTB specific mitogen levels and still found no association with HIV (AOR (95% CI), p-value) 0.91 (0.31–2.64), p = 0.87).

We further carried out another sub analysis to assess how HIV infection could have affected IP-10 production among the household contact in the different age groups. Eleven of the contacts with HIV were above 18 years, and there was no difference in either the MTB-specific IP-10 or mitogen specific responses between the HIV negative and HIV positive in this group (p = 0.24 and p = 0.74 respectively) (Fig 4C and 4D).

### Relationship between IP-10 and IFNγ responses and TST diameters at baseline and follow up

**Fig 5:**
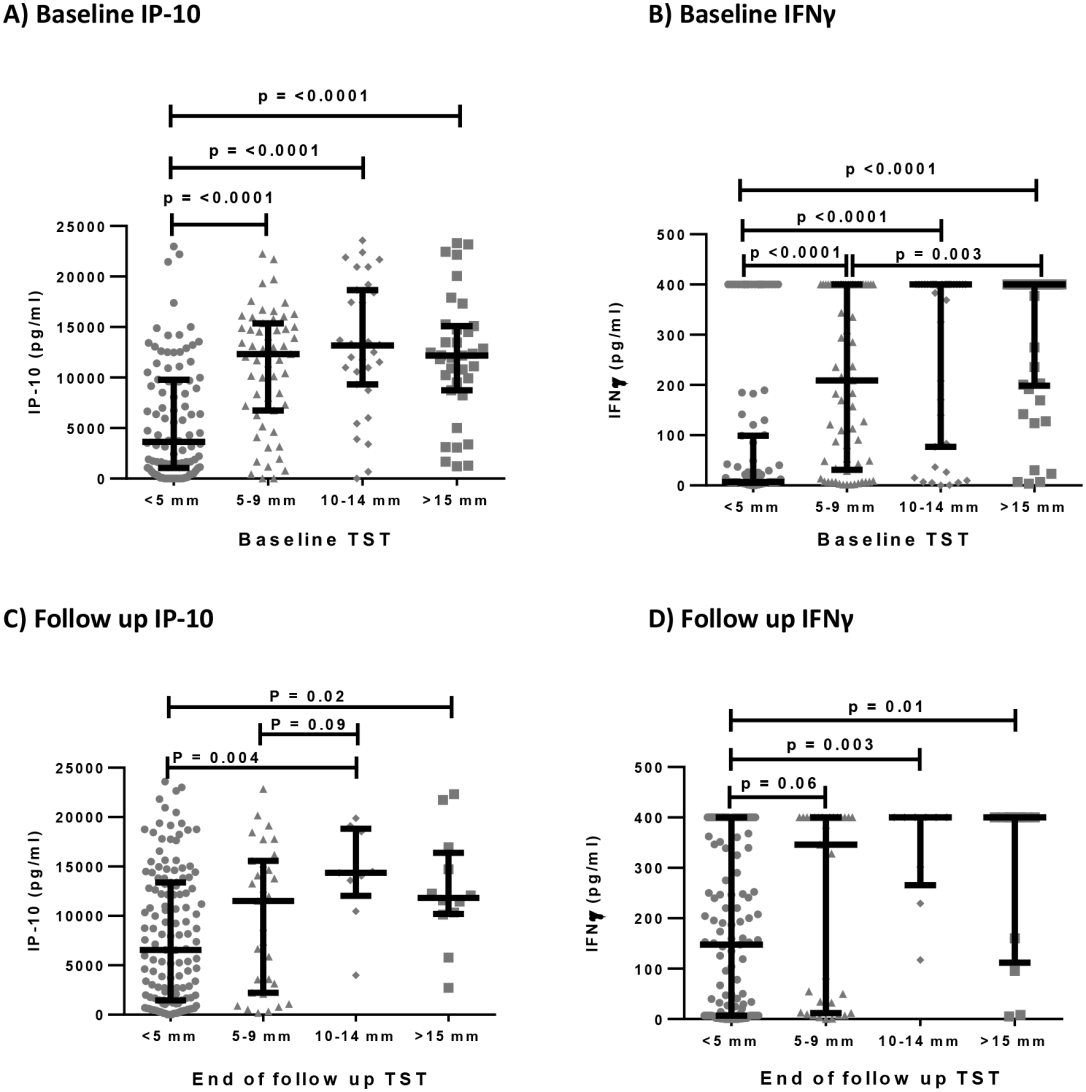
The relationship between TST diameter and IP-10 or IFNγ production in QuantiFERON test supernatants. Bar graphs show baseline and end of follow up median comparisons of IP-10 levels (A and C) and IFNγ levels from the QFN test (B and D) with TST diameters. Comparisons were tested using the Mann-Whitney *U* test.

We assessed whether IP-10 and IFNγ levels from the QFN test were associated with TST diameter (categorised as <5mm, 5-9mm, 10-14mm, >15mm) at baseline and six months later. The contacts with a TST diameter of <5mm produced the lowest concentrations of IP-10 (Fig 5A) and IFNγ (Fig 5B) at baseline compared with contacts with larger TST diameters. The pattern was similar at the end of six months of follow up (Fig 5C and 5D).

### High baseline IP-10 responses are associated with conversion of the TST test from negative to positive

**Fig 6:**
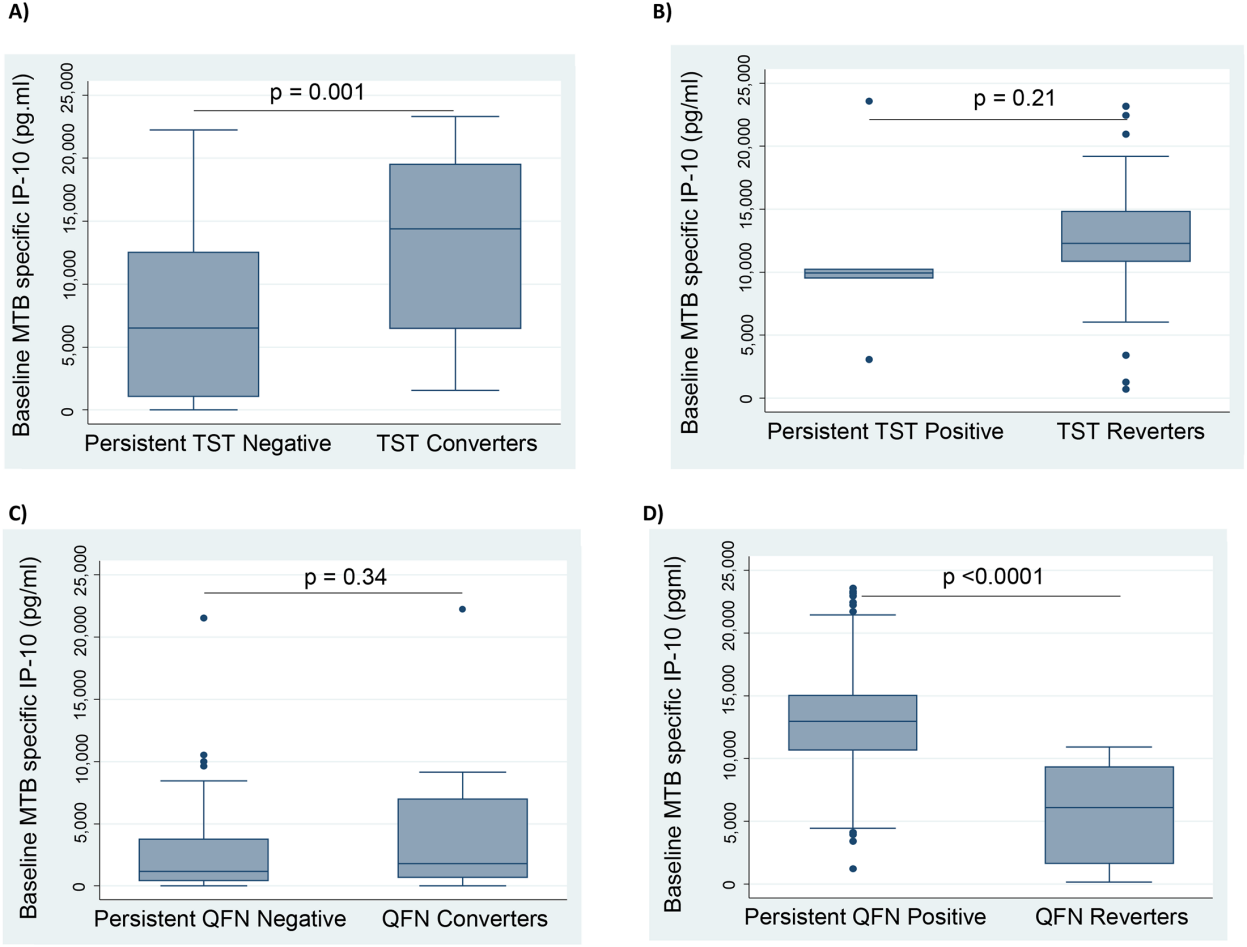
Assessing IP-10 concentrations among household contacts that converted their TST/QFN test results from negative to positive or reverted their TST/QFN test results from positive to negative. Box graphs comparing median baseline MTB specific IP-10 levels among A) TST converters, B) TST reverters, C) QFN converters, D) QFN reverters. IP-10 was measured in QFT supernatants in contacts at baseline. Contacts with TST conversions, n = 16; or QFN conversions, n = 14 were compared with contacts who were persistently TST negative, n = 98 or persistently QFN negative, n = 46. Contacts with TST reversions, n = 36 or QFN reversions, n = 10 were compared with contacts who were persistently TST positive, n = 5 or persistently QFN positive, n = 104. Mann-Whitney *U* test A) p = 0.001, B) p = 0.21, C) p = 0.34, D) p <0.0001. Pairwise comparisons were made using the Mann-Whitney *U* test.

In this study, 52% (36/69) of the contacts had TST reversions and 10% (16/168) TST conversions between baseline at six-months follow-up, while there were 7% (10/154) QFN reversions and 17% (14/83) QFN conversions. The baseline concentrations of IP-10 were significantly higher among the baseline TST negative contacts who proceeded to TST conversion, compared to those who did not (Fig 6A), but there was no difference in baseline IP-10 among the contacts with or without TST reversion (Fig 6B). Contrarily, there was no difference in baseline IP-10 among QFN-negatives who proceeded to QFN conversion, compared to those who did not (Fig 6C), whereas among QFN positive contacts, those who reverted had lower baseline concentrations of IP-10 than those who did not (Fig 6D).

## Discussion

In this prospective study, we analysed whole blood QFN culture supernatants at baseline and after six months for IP-10 responses and found that IP-10 is potentially a good diagnostic marker for LTBI and active TB disease [31]. The biomarker was easy to detect, being produced at very high levels even in children and in individuals with HIV infection [34, 47, 48]. A study by Petrone *et al*., in the same setting in Uganda were not able to accurately diagnose active TB in children using plasma IP-10. In addition, Holm *et al*., carried out a study in a similar setting of a high TB burden and found that IP-10 performed well in diagnosing TB disease in adults but not in children [43]. Both these studies showed low sensitivities of IP-10 in children. However, the children in the studies either had confirmed or probable TB disease, unlike the children in our study who were healthy contacts. There was also heavy influence of HIV infection in their findings, which was not demonstrated in our study. Furthermore, the children in our study showed no signs or symptoms of severe malnutrition. The active TB cases in this study had very low IP-10 responses following stimulation with the mitogen PHA compared to that of contacts [49]. This could be as a result of immunosuppression by MTB or from HIV co-infection which may have led to low lymphocyte counts. However, we found that there was no association between the HIV status of either the TB cases or contacts and their MTB specific IP-10 levels.

The air borne nature of MTB transmission means that closer proximity to the index case and longer duration interacting with the index case increases the risk of TB infectiousness [3, 50]. Therefore a good test to diagnose LTBI or active TB should correlate well with the extent of TB exposure and would not be influenced by the BCG immunisation status of the individual resulting in false positive results as seen with the TST [2, 5, 26, 51]. In this study, MTB specific IP-10 was strongly associated with TB exposure making it comparable to TST and QFN in diagnosing LTBI, and was not influenced by the presence of a BCG scar [34, 52].

Effects of BCG immunisation on TST positivity have been previously reported [18, 20, 25] and could have contributed to the discordance between TST and QFN especially when BCG is given at birth. The effects of BCG at birth can last for at least as long as 15 years [27]. In BCG unvaccinated populations, there is better concordance between TST and the IGRA tests [23]. The effect of BCG on TST depends on the timing of the vaccine, the frequency at which it is given, the type of BCG strain used in the vaccine, and the time since vaccination [27]. We found higher MTB-specific IP-10 concentrations and TST positivity in those with lack of a BCG scar, suggesting that BCG was conferring some protection in our setting [53, 54].

We assessed the performance of IP-10 to determine whether there was an added benefit of using IP-10 as a potential biomarker in the IGRA test and found that IP-10 was comparable to IFNγ [34]. IP-10 has the advantage of being easy to detect even in children or in persons with HIV infection unlike the IGRA where there is evidence of many indeterminate results in the same populations [41]. In this study, there was good agreement between IP-10 and QFN, moderate agreement between IP-10 and TST and poor agreement between QFN and TST. This contrasts with a study by Pai, *et al*., in health care workers in India which is a high TB endemic setting, which reported high TST and QFN agreement [28].

Similar to IFNγ in the IGRA, IP-10 was not able to differentiate between latent infection and active TB disease [55–57]. Recent evidence by Wergeland, *et al*., demonstrated how IP-10 differentiated between active TB cases and LTBI or QFN negative controls [58]. The differences in our findings could be explained by the differences in study design and populations. Their study was carried out in a low TB endemic area, where they included only adult patients, they defined LTBI based on only a positive QFN, and used multiplex cytokine assay to detect IP-10 which had much lower cut offs for detection. While our study was carried out in a high TB endemic area, the study population included children and adults, we defined LTBI as positive QFN and TST, and used an ELISA assay to detect IP-10 at higher cut offs. We further explored whether MTB specific to mitogen specific IP-10 ratios could discriminate individuals with LTBI from those with active TB. Despite the active TB cases having a slightly higher ratio, when we performed the ROC analyses in the study subjects above 18 years, the AUC was 0.60 which showed that the MTB specific to mitogen specific ratios could not be used to differentiate between LTBI and active TB. However, when we performed this analysis in the HIV negative population, we obtained an AUC of 0.7 which was able to discriminate between LTBI and active TB in this sub population. This finding is similar to work carried out by Jeong, *et al*., who found that MTB specific to mitogen induced IP-10 ratios could be used to differentiate between latent infection and active TB disease irrespective of HIV status [49]. In the Jeong study, MTB specific to mitogen specific IP-10 ratios could discriminate between LTBI and active TB in individuals irrespective of their HIV status, while in our study, they could only discriminate between the two disease states among HIV negative individuals. It was also of interest to note that despite the location of the study area being in a low TB burden setting, the active TB cases produced very high quantities of IP-10, almost four time as high as what the active TB cases in our study (a high TB endemic setting) produced. This could have contributed to the high MTB specific to mitogen induced IP-10 ratios, and the ability to achieve an AUC well above 0.8, thus allowing the ratio to adequately discriminate between LTBI and active TB. They however used a multiplex cytokine assay to detect for IP-10 responses.

TST or DTH responses do not always correlate with IFNγ responses [59]. A low TST response with high IGRA responses may also be attributed to constant exposure to non-tuberculosis mycobacteria which may cause anergy [26, 60] or boosting of DTH immune responses from recent mycobacterial exposure which is easily detected by the IGRA and not TST [60]. Our household cohort had many children who presented with TST negative results. The findings were similar to those reported by Mudido, *et al*., among Ugandan children despite BCG immunisation at birth [29]. This could be an effect of HIV infection [61] but most of the contacts in our study were HIV negative. It could also be as a result of malnutrition [4] though none of the contacts presented with symptoms or signs of severe malnutrition. There have been suggestions that differences in human leucocyte antigen (HLA) among individuals can also lead to differences in DTH or IFNγ immune responses [62]. Another possible reason for the differences in findings between studies could be related to the different manufacturers of tuberculin used, with RT23 2TU or 1TU giving more positive TST results than 5TU PPD from other sources [27]. We used RT23 2TU in our study but still detected few positive responses using TST. A study by Floyd, *et al*., showed how TST results varied with latitude [63] and there was a reduction in TST responses in the tropics. There could also be misclassification of BCG status secondary to the use of the presence of a BCG scar as a surrogate [28], although over 80% of the contacts had a BCG scar. A study by Soysal, *et al*., found that presence of a BCG scar was associated with reduced infection defined using IGRA but not TST to test for MTB infection [64]. In our study, contacts with a BCG scar had lower responses on all the three tests. Another possibility might be prenatal exposure to TB antigens in this highly endemic area, resulting in tolerance.

When we compared IP-10 response with the different TST diameters, we found that the IP-10 response increased with increasing TST diameters. We further investigated the relationship of MTB specific IP-10 response with QFN and TST test reversions and conversions. Our study had a high number of TST reversions which is not uncommon [65]. The baseline MTB specific IP-10 response was marked among the contacts who converted their TST from negative to positive. This supports the ability of IP-10 to diagnose ‘recent TB infection’ in individuals classified as having LTBI using the TST, and could suggest that high MTB specific IP-10 responses in absence of the TST may point towards early infection. Further still, we think that such high IP-10 responses might be a good discriminator of established MTB infections, as opposed to transient MTB-specific sensitisation. This could help identify people most likely to benefit from chemoprophylaxis with isoniazid preventive therapy. A study by Buchwald, *et al*., also suggested IP-10 as a potential marker of recent infection [66].

Interestingly, we also found that the contacts who reverted their QFN test produced lower MTB specific IP-10 responses than those that had a persistent positive QFN test. Thus a low MTB specific IP-10 response in the presence of a negative QFN test may suggest a threshold of exposure less likely to establish infection in such individuals.

The main limitation of this study was the absence of a gold standard for diagnosing LTBI and this is a challenge for the development of any diagnostic test for LTBI. We used the combination of TST and QFN positivity to define LTBI and assess the performance of IP-10. The other possible limitation could have been the choice of using QFN supernatants to assess for IP-10 responses, although QFN samples have been used before to measure IP-10 responses with similar results [40]. The inability to measure the effect of NTM on the three test responses is another possible limitation. Lastly, IP-10 expression has been shown to be increased in a number of infections including TB disease [34], which could affect its specificity as a biomarker [67], but we however, found IP-10 responses to MTB-specific antigens to be highly specific for TB infection and disease. A review by Ruhwald, *et al*., showed variability of findings on the potential use of IP-10 as a biomarker in diagnosis of TB disease, as being influenced by differences in dilution of samples, type of samples used, assays to measure IP-10, and diagnostic cut-offs used [34]. Our study however was able to demonstrate that IP-10 has a high potential of being used to diagnose latent tuberculosis and in addition, it could be used as a marker of recent infection with MTB and of individuals particularly likely to benefit from isoniazid preventive therapy.

## Conclusion

High IP-10 concentrations were measured in QFN supernatants from latently infected contacts compared to uninfected contacts or active TB cases. Therefore, IP-10 can be used to diagnose LTBI because it is easily detectable though active TB disease needs to be ruled out. The baseline prevalence of LTBI using TST and QFN were 32% and 65% respectively. An IP-10 cut-off of 8,239 pg/ml maximised sensitivity/specificity compared to the QFN^+^TST^+^ standard. Using this cut-off, the baseline LTBI prevalence was 55%. IP-10 performed well in differentiating contacts with either latent or active TB from those who were uninfected but was not able to differentiate latent infection from active disease except when MTB specific to mitogen specific ratios were used in HIV negative adults. However, we also demonstrated the ability of IP-10 to diagnose ‘recent TB infection’ and hence transmission in individuals classified as having LTBI using the TST. Such strong IP-10 producers would be a likely target group to benefit from chemoprophylaxis.

## Supporting Information

S1 Fig Correlation between IP-10 and interferon gamma (IFNγ) in the QFN test. Scatter graphs showing the correlation between IP-10 levels and IFNγ among A) household contacts, B) index cases.
(TIF)

## References

[1] Corbett EL, Watt CJ, Walker N, Maher D, Williams BG, Raviglione MC, et al The growing burden of tuberculosis: global trends and interactions with the HIV epidemic. Arch Intern Med. 2003;163(9):1009–21. Epub 2003/05/14. 10.1001/archinte.163.9.1009 12742798

[2] Richeldi L. An update on the diagnosis of tuberculosis infection. Am J Respir Crit Care Med. 2006;174(7):736–42. Epub 2006/06/27. 10.1164/rccm.200509-1516PP . 16799073

[3] Lalvani A. Spotting latent infection: the path to better tuberculosis control. Thorax. 2003;58(11):916–8. Epub 2003/10/31. 14586040 10.1136/thorax.58.11.916 PMC1746498

[4] Jasmer RM, Nahid P, Hopewell PC. Clinical practice. Latent tuberculosis infection. N Engl J Med. 2002;347(23):1860–6. Epub 2002/12/06. 10.1056/NEJMcp021045 . 12466511

[5] Lalvani A. Diagnosing tuberculosis infection in the 21st century: new tools to tackle an old enemy. Chest. 2007;131(6):1898–906. Epub 2007/06/15. 131/6/1898 [pii] 10.1378/chest.06-2471 . 17565023

[6] Pai M, Zwerling A, Menzies Systematic review: T-cell-based assays for the diagnosis of latent tuberculosis infection: an update. Ann Intern Med. 2008;8 5;149(3):177–84. 18593687 10.7326/0003-4819-149-3-200808050-00241 PMC2951987

[7] Lange C, Mori T. Advances in the diagnosis of tuberculosis. Respirology. 2010;15(2):220–40. Epub 2010/03/05. 10.1111/j.1440-1843.2009.01692.x . 20199641

[8] Menzies D. Interpretation of repeated tuberculin tests. Boosting, conversion, and reversion. Am J Respir Crit Care Med. 1999;159(1):15–21. Epub 1999/01/05. 10.1164/ajrccm.159.1.9801120 . 9872812

[9] Menzies D, Pai M, Comstock G. Meta-analysis: new tests for the diagnosis of latent tuberculosis infection: areas of uncertainty and recommendations for research. Ann Intern Med. 2007;146(5):340–54. Epub 2007/03/07. 146/5/340 [pii]. . 17339619 10.7326/0003-4819-146-5-200703060-00006

[10] Cole ST, Brosch R, Parkhill J, Garnier T, Churcher C, Harris D, et al Deciphering the biology of Mycobacterium tuberculosis from the complete genome sequence. Nature. 1998;393(6685):537–44. Epub 1998/06/20. 10.1038/31159 . 9634230

[11] Harboe M, Oettinger T, Wiker HG, Rosenkrands I, Andersen P. Evidence for occurrence of the ESAT-6 protein in Mycobacterium tuberculosis and virulent Mycobacterium bovis and for its absence in Mycobacterium bovis BCG. Infect Immun. 1996;64(1):16–22. Epub 1996/01/01. 8557334 10.1128/iai.64.1.16-22.1996 PMC173721

[12] Sorensen AL, Nagai S, Houen G, Andersen P, Andersen AB. Purification and characterization of a low-molecular-mass T-cell antigen secreted by Mycobacterium tuberculosis. Infect Immun. 1995;63(5):1710–7. Epub 1995/05/01. 7729876 10.1128/iai.63.5.1710-1717.1995 PMC173214

[13] Berthet FX, Rasmussen PB, Rosenkrands I, Andersen P, Gicquel B. A Mycobacterium tuberculosis operon encoding ESAT-6 and a novel low-molecular-mass culture filtrate protein (CFP-10). Microbiology. 1998;144 (Pt 11):3195–203. Epub 1998/12/10. . 9846755 10.1099/00221287-144-11-3195

[14] Behr MA, Wilson MA, Gill WP, Salamon H, Schoolnik GK, Rane S, et al Comparative genomics of BCG vaccines by whole-genome DNA microarray. Science. 1999;284(5419):1520–3. Epub 1999/05/29. . 10348738 10.1126/science.284.5419.1520

[15] Mahairas GG, Sabo PJ, Hickey MJ, Singh DC, Stover CK. Molecular analysis of genetic differences between Mycobacterium bovis BCG and virulent M. bovis. Journal of bacteriology. 1996;178(5):1274–82. Epub 1996/03/01. 8631702 10.1128/jb.178.5.1274-1282.1996 PMC177799

[16] Andersen P, Munk ME, Pollock JM, Doherty TM. Specific immune-based diagnosis of tuberculosis. Lancet. 2000;356(9235):1099–104. Epub 2000/09/29. . 11009160 10.1016/s0140-6736(00)02742-2

[17] Mori T, Sakatani M, Yamagishi F, Takashima T, Kawabe Y, Nagao K, et al Specific detection of tuberculosis infection: an interferon-gamma-based assay using new antigens. Am J Respir Crit Care Med. 2004;170(1):59–64. Epub 2004/04/03. 10.1164/rccm.200402-179OC . 15059788

[18] Ewer K, Deeks J, Alvarez L, Bryant G, Waller S, Andersen P, et al Comparison of T-cell-based assay with tuberculin skin test for diagnosis of Mycobacterium tuberculosis infection in a school tuberculosis outbreak. Lancet. 2003;361(9364):1168–73. Epub 2003/04/11. S0140-6736(03)12950-9 [pii] 10.1016/S0140-6736(03)12950-9 . 12686038

[19] Nkurunungi G, Lutangira JE, Lule SA, Akurut H, Kizindo R, Fitchett JR, et al Determining Mycobacterium tuberculosis infection among BCG-immunised Ugandan children by T-SPOT.TB and tuberculin skin testing. PLOS One. 2012;7(10):e47340 Epub 2012/10/19. 10.1371/journal.pone.0047340 23077594 PMC3471887

[20] Kang YA, Lee HW, Yoon HI, Cho B, Han SK, Shim YS, et al Discrepancy between the tuberculin skin test and the whole-blood interferon gamma assay for the diagnosis of latent tuberculosis infection in an intermediate tuberculosis-burden country. JAMA. 2005;293(22):2756–61. Epub 2005/06/09. 293/22/2756 [pii] 10.1001/jama.293.22.2756 . 15941805

[21] Connell TG, Curtis N, Ranganathan SC, Buttery JP. Performance of a whole blood interferon gamma assay for detecting latent infection with Mycobacterium tuberculosis in children. Thorax. 2006;61(7):616–20. Epub 2006/04/08. 10.1136/thx.2005.048033 16601088 PMC2104654

[22] Mandalakas AM, Hesseling AC, Chegou NN, Kirchner HL, Zhu X, Marais BJ, et al High level of discordant IGRA results in HIV-infected adults and children. Int J Tuberc Lung Dis. 2008;12(4):417–23. Epub 2008/03/29. . 18371268

[23] Diel R, Ernst M, Doscher G, Visuri-Karbe L, Greinert U, Niemann S, et al Avoiding the effect of BCG vaccination in detecting Mycobacterium tuberculosis infection with a blood test. Eur Respir J. 2006;28(1):16–23. Epub 2006/02/17. 10.1183/09031936.06.00107005 . 16481383

[24] Harada N, Nakajima Y, Higuchi K, Sekiya Y, Rothel J, Mori T. Screening for tuberculosis infection using whole-blood interferon-gamma and Mantoux testing among Japanese healthcare workers. Infection control and hospital epidemiology: the official journal of the Society of Hospital Epidemiologists of America. 2006;27(5):442–8. Epub 2006/05/04. 10.1086/504358 . 16671023

[25] Brock I, Weldingh K, Lillebaek T, Follmann F, Andersen P. Comparison of tuberculin skin test and new specific blood test in tuberculosis contacts. Am J Respir Crit Care Med. 2004;170(1):65–9. Epub 2004/04/17. 10.1164/rccm.200402-232OC . 15087297

[26] Hill PC, Brookes RH, Fox A, Fielding K, Jeffries DJ, Jackson-Sillah D, et al Large-scale evaluation of enzyme-linked immunospot assay and skin test for diagnosis of Mycobacterium tuberculosis infection against a gradient of exposure in The Gambia. Clin Infect Dis. 2004;38(7):966–73. Epub 2004/03/23.[pii]. . 15034828 10.1086/382362

[27] Wang L, Turner MO, Elwood RK, Schulzer M, FitzGerald JM. A meta-analysis of the effect of Bacille Calmette Guerin vaccination on tuberculin skin test measurements. Thorax. 2002;57(9):804–9. Epub 2002/08/30. 12200526 10.1136/thorax.57.9.804 PMC1746436

[28] Pai M, Gokhale K, Joshi R, Dogra S, Kalantri S, Mendiratta DK, et al Mycobacterium tuberculosis infection in health care workers in rural India: comparison of a whole-blood interferon gamma assay with tuberculin skin testing. JAMA. 2005;293(22):2746–55. Epub 2005/06/09. 293/22/2746 [pii] 10.1001/jama.293.22.2746 . 15941804

[29] Mudido PM, Guwatudde D, Nakakeeto MK, Bukenya GB, Nsamba D, Johnson JL, et al The effect of bacille Calmette-Guerin vaccination at birth on tuberculin skin test reactivity in Ugandan children. Int J Tuberc Lung Dis. 1999;3(10):891–5. Epub 1999/10/19. . 10524586

[30] Chen YC, Chin CH, Liu SF, Wu CC, Tsen CC, Wang YH, et al Prognostic values of serum IP-10 and IL-17 in patients with pulmonary tuberculosis. Disease markers. 2011;31(2):101–10. Epub 2011/09/08. 10.3233/DMA-2011-0808 21897004 PMC3826581

[31] Chegou NN, Heyckendorf J, Walzl G, Lange C, Ruhwald M. Beyond the IFN-gamma horizon: biomarkers for immunodiagnosis of infection with Mycobacterium tuberculosis. Eur Respir J. 2014;43(5):1472–86. Epub 2013/12/07. 10.1183/09031936.00151413 . 24311770

[32] Farber JM. Mig and IP-10: CXC chemokines that target lymphocytes. J Leukoc Biol. 1997;61(3):246–57. Epub 1997/03/01. . 9060447

[33] Luster AD, Unkeless JC, Ravetch JV. Gamma-interferon transcriptionally regulates an early-response gene containing homology to platelet proteins. Nature. 1985;315(6021):672–6. Epub 1985/06/20. . 3925348 10.1038/315672a0

[34] Ruhwald M, Aabye MG, Ravn P. IP-10 release assays in the diagnosis of tuberculosis infection: current status and future directions. Expert Rev Mol Diagn. 2012;12(2):175–87. Epub 2012/03/01. 10.1586/erm.11.97 . 22369377

[35] Dufour JH, Dziejman M, Liu MT, Leung JH, Lane TE, Luster AD. IFN-gamma-inducible protein 10 (IP-10; CXCL10)-deficient mice reveal a role for IP-10 in effector T cell generation and trafficking. J Immunol. 2002;168(7):3195–204. Epub 2002/03/22. . 11907072 10.4049/jimmunol.168.7.3195

[36] Ferrero E, Biswas P, Vettoretto K, Ferrarini M, Uguccioni M, Piali L, et al Macrophages exposed to Mycobacterium tuberculosis release chemokines able to recruit selected leucocyte subpopulations: focus on gammadelta cells. Immunology. 2003;108(3):365–74. Epub 2003/02/27. 12603603 10.1046/j.1365-2567.2003.01600.x PMC1782907

[37] Orme IM, Cooper AM. Cytokine/chemokine cascades in immunity to tuberculosis. Immunol Today. 1999;20(7):307–12. Epub 1999/06/24. . 10379048 10.1016/s0167-5699(98)01438-8

[38] Kaplan G, Luster AD, Hancock G, Cohn ZA. The expression of a gamma interferon-induced protein (IP-10) in delayed immune responses in human skin. J Exp Med. 1987;166(4):1098–108. Epub 1987/10/01. 2443597 10.1084/jem.166.4.1098 PMC2188712

[39] Azzurri A, Sow OY, Amedei A, Bah B, Diallo S, Peri G, et al IFN-gamma-inducible protein 10 and pentraxin 3 plasma levels are tools for monitoring inflammation and disease activity in Mycobacterium tuberculosis infection. Microbes Infect. 2005;7(1):1–8. Epub 2005/02/18. 10.1016/j.micinf.2004.09.004 . 15716076

[40] Ruhwald M, Bjerregaard-Andersen M, Rabna P, Kofoed K, Eugen-Olsen J, Ravn P. CXCL10/IP-10 release is induced by incubation of whole blood from tuberculosis patients with ESAT-6, CFP10 and TB7.7. Microbes Infect. 2007;9(7):806–12. Epub 2007/05/30. S1286-4579(07)00101-3 [pii] 10.1016/j.micinf.2007.02.021 . 17533146

[41] Mandalakas AM, Kirchner HL, Walzl G, Gie RP, Schaaf HS, Cotton MF, et al Optimizing the Detection of Recent Tuberculosis Infection in Children in a High Tuberculosis-HIV Burden Setting. Am J Respir Crit Care Med. 2015;191(7):820–30. Epub 2015/01/27. 10.1164/rccm.201406-1165OC . 25622087 PMC4407483

[42] Lighter J, Rigaud M, Huie M, Peng CH, Pollack H. Chemokine IP-10: an adjunct marker for latent tuberculosis infection in children. Int J Tuberc Lung Dis. 2009;13(6):731–6. . 19460249

[43] Holm LL, Rose MV, Kimaro G, Bygbjerg IC, Mfinanga SG, Ravn P, et al A comparison of interferon-gamma and IP-10 for the diagnosis of tuberculosis. Pediatrics. 2014;134(6):e1568–75. Epub 2014/11/26. 10.1542/peds.2014-1570 . 25422019

[44] Petrone L, Cannas A, Aloi F, Nsubuga M, Sserumkuma J, Nazziwa RA, et al Blood or Urine IP-10 Cannot Discriminate between Active Tuberculosis and Respiratory Diseases Different from Tuberculosis in Children. BioMed research international. 2015;2015:589471 10.1155/2015/589471 26346028 PMC4540955

[45] Biraro IA, Egesa M, Toulza F, Levin J, Cose S, Joloba M, et al Impact of co-infections and BCG immunisation on immune responses among household contacts of tuberculosis patients in a Ugandan cohort. PLOS One. 2014;9(11):e111517 Epub 2014/11/06. 10.1371/journal.pone.0111517 25372043 PMC4221037

[46] BD Bioscience O. Human IP-10 ELISA Set: Technical Data Sheet 2011;(Cat. No.550926).

[47] Ruhwald M, Petersen J, Kofoed K, Nakaoka H, Cuevas LE, Lawson L, et al Improving T-cell assays for the diagnosis of latent TB infection: potential of a diagnostic test based on IP-10. PLOS One. 2008;3(8):e2858 Epub 2008/08/07. 10.1371/journal.pone.0002858 18682747 PMC2483344

[48] Whittaker E, Gordon A, Kampmann B. Is IP-10 a better biomarker for active and latent tuberculosis in children than IFNgamma? PLOS One. 2008;3(12):e3901 Epub 2008/12/10. 10.1371/journal.pone.0003901 19065267 PMC2588495

[49] Jeong YH, Hur YG, Lee H, Kim S, Cho JE, Chang J, et al Discrimination between active and latent tuberculosis based on ratio of antigen-specific to mitogen-induced IP-10 production. J Clin Microbiol. 2015;53(2):504–10. Epub 2014/11/28. 10.1128/JCM.02758-14 25428147 PMC4298513

[50] Lienhardt C, Fielding K, Sillah J, Tunkara A, Donkor S, Manneh K, et al Risk factors for tuberculosis infection in sub-Saharan Africa: a contact study in The Gambia. Am J Respir Crit Care Med. 2003;168(4):448–55. Epub 2003/05/30. 10.1164/rccm.200212-1483OC 200212-1483OC [pii]. . 12773322

[51] Lalvani A, Pathan AA, Durkan H, Wilkinson KA, Whelan A, Deeks JJ, et al Enhanced contact tracing and spatial tracking of Mycobacterium tuberculosis infection by enumeration of antigen-specific T cells. Lancet. 2001;23 6;357(9273):2017–21. 11438135 10.1016/S0140-6736(00)05115-1

[52] Pai M, Riley LW, Colford JM Jr. Interferon-gamma assays in the immunodiagnosis of tuberculosis: a systematic review. Lancet Infect Dis. 2004;4(12):761–76. Epub 2004/11/30. 10.1016/S1473-3099(04)01206-X . 15567126

[53] Roy A, Eisenhut M, Harris RJ, Rodrigues LC, Sridhar S, Habermann S, et al Effect of BCG vaccination against Mycobacterium tuberculosis infection in children: systematic review and meta-analysis. BMJ. 2014;349:g4643 Epub 2014/08/07. 10.1136/bmj.g4643 25097193 PMC4122754

[54] Eisenhut M, Paranjothy S, Abubakar I, Bracebridge S, Lilley M, Mulla R, et al BCG vaccination reduces risk of infection with Mycobacterium tuberculosis as detected by gamma interferon release assay. Vaccine. 2009;27(44):6116–20. Epub 2009/09/01. 10.1016/j.vaccine.2009.08.031 . 19715782

[55] Goletti D, Raja A, Ahamed Kabeer BS, Rodrigues C, Sodha A, Butera O, et al IFN-gamma, but not IP-10, MCP-2 or IL-2 response to RD1 selected peptides associates to active tuberculosis. J Infect. 2010;61(2):133–43. Epub 2010/05/18. S0163-4453(10)00133-7 [pii] 10.1016/j.jinf.2010.05.002 . 20470822

[56] Lalvani A, Millington KA. T-cell interferon-gamma release assays: can we do better? Eur Respir J. 2008;32(6):1428–30. Epub 2008/12/02. 32/6/1428 [pii] 10.1183/09031936.00148308 . 19043006

[57] Vanini V, Petruccioli E, Gioia C, Cuzzi G, Orchi N, Rianda A, et al IP-10 is an additional marker for tuberculosis (TB) detection in HIV-infected persons in a low-TB endemic country. J Infect. 2012;65(1):49–59. 10.1016/j.jinf.2012.03.017 . 22465752

[58] Wergeland I, Pullar N, Assmus J, Ueland T, Tonby K, Feruglio S, et al IP-10 differentiates between active and latent tuberculosis irrespective of HIV status and declines during therapy. J Infect. 2015 Epub 2015/01/20. 10.1016/j.jinf.2014.12.019 . 25597826

[59] Hoft DF, Brown RM, Belshe RB. Mucosal bacille calmette-Guerin vaccination of humans inhibits delayed-type hypersensitivity to purified protein derivative but induces mycobacteria-specific interferon-gamma responses. Clin Infect Dis. 2000;30 Suppl 3:S217–22. Epub 2000/06/30. 10.1086/313864 . 10875787

[60] Black GF, Weir RE, Floyd S, Bliss L, Warndorff DK, Crampin AC, et al BCG-induced increase in interferon-gamma response to mycobacterial antigens and efficacy of BCG vaccination in Malawi and the UK: two randomised controlled studies. Lancet. 2002;359(9315):1393–401. Epub 2002/04/30. 10.1016/S0140-6736(02)08353-8 . 11978337

[61] Weinfurter P, Blumberg HM, Goldbaum G, Royce R, Pang J, Tapia J, et al Predictors of discordant tuberculin skin test and QuantiFERON(R)-TB Gold In-Tube results in various high-risk groups. Int J Tuberc Lung Dis. 2011;15(8):1056–61. Epub 2011/07/12. 10.5588/ijtld.10.0650 . 21740668

[62] Arend SM, Geluk A, van Meijgaarden KE, van Dissel JT, Theisen M, Andersen P, et al Antigenic equivalence of human T-cell responses to Mycobacterium tuberculosis-specific RD1-encoded protein antigens ESAT-6 and culture filtrate protein 10 and to mixtures of synthetic peptides. Infect Immun. 2000;68(6):3314–21. Epub 2000/05/19. 10816479 10.1128/iai.68.6.3314-3321.2000 PMC97589

[63] Floyd S, Ponnighaus JM, Bliss L, Nkhosa P, Sichali L, Msiska G, et al Kinetics of delayed-type hypersensitivity to tuberculin induced by bacille Calmette-Guerin vaccination in northern Malawi. J Infect Dis. 2002;186(6):807–14. Epub 2002/08/29. 10.1086/342416 . 12198615

[64] Soysal A, Millington KA, Bakir M, Dosanjh D, Aslan Y, Deeks JJ, et al Effect of BCG vaccination on risk of Mycobacterium tuberculosis infection in children with household tuberculosis contact: a prospective community-based study. Lancet. 2005;366(9495):1443–51. Epub 2005/10/26. S0140-6736(05)67534-4 [pii] 10.1016/S0140-6736(05)67534-4 . 16243089

[65] Fine PE, Bruce J, Ponnighaus JM, Nkhosa P, Harawa A, Vynnycky E. Tuberculin sensitivity: conversions and reversions in a rural African population. Int J Tuberc Lung Dis. 1999;3(11):962–75. Epub 1999/12/10. . 10587318

[66] Buchwald UK, Adetifa IM, Bottomley C, Owiafe PK, Donkor S, Bojang AL, et al Broad adaptive immune responses to M. tuberculosis antigens precede TST conversion in tuberculosis exposed household contacts in a TB-endemic setting. PLOS One. 2014;9(12):e116268 Epub 2014/12/31. 10.1371/journal.pone.0116268 25549338 PMC4280211

[67] Syed Ahamed Kabeer B, Raman B, Thomas A, Perumal V, Raja A. Role of QuantiFERON-TB gold, interferon gamma inducible protein-10 and tuberculin skin test in active tuberculosis diagnosis. PLOS One. 2010;5(2):e9051 Epub 2010/02/09. 10.1371/journal.pone.0009051 20140219 PMC2816212

