# Supplementary figures and images for: The Use of Interferon Gamma Inducible Protein 10 as a Potential Biomarker in the Diagnosis of Latent Tuberculosis Infection in Uganda
Source: PLoS One. 2016 Jan 15;11(1):e0146098. doi: 10.1371/journal.pone.0146098 (PMC4714877; doi:10.1371/journal.pone.0146098)

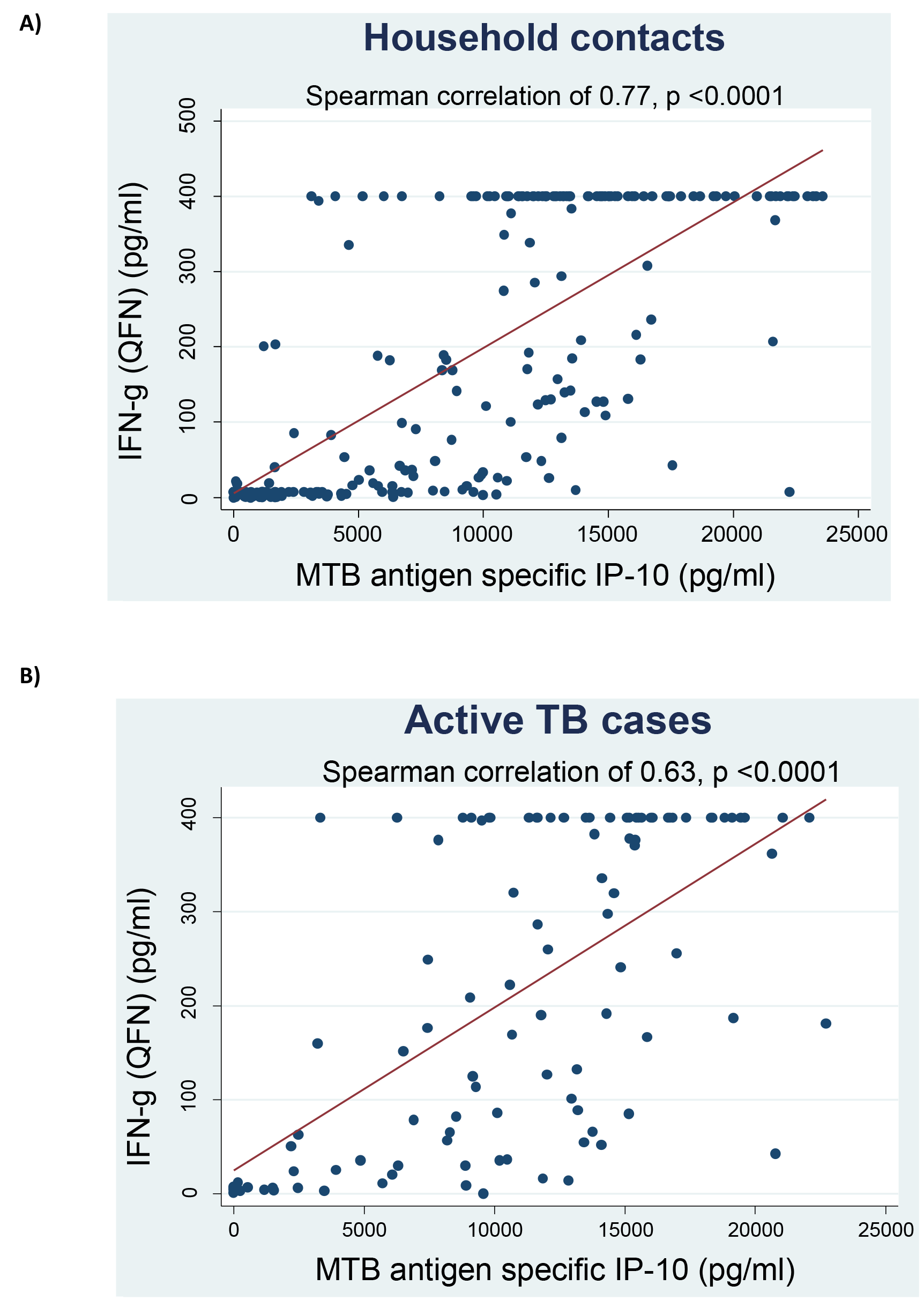

Supplement: S1 Fig — Scatter graphs showing the correlation between IP-10 levels and IFNγ among A) household contacts, B) index cases. (TIF) [file pone.0146098.s001.tif]
